# Supplementary material for: Inhibiting the integrated stress response pathway prevents aberrant chondrocyte differentiation thereby alleviating chondrodysplasia
Source: eLife. 2018 Jul 19;7:e37673. doi: 10.7554/eLife.37673 (PMC6053305; doi:10.7554/eLife.37673)
Supplement: Transparent reporting form [file elife-37673-transrepform.pdf]

eLife's transparent reporting form

We encourage authors to provide detailed information *within their submission* to facilitate the interpretation and replication of experiments. Authors can upload supporting documentation to indicate the use of appropriate reporting guidelines for health-related research (see EQUATOR Network), life science research (see the BioSharing Information Resource), or the ARRIVE guidelines for reporting work involving animal research. Where applicable, authors should refer to any relevant reporting standards documents in this form.

If you have any questions, please consult our Journal Policies and/or contact us: editorial@elifesciences.org.

Sample-size estimation

- You should state whether an appropriate sample size was computed when the study was being designed
- You should state the statistical method of sample size computation and any required assumptions
- If no explicit power analysis was used, you should describe how you decided what sample (replicate) size (number) to use

Please outline where this information can be found within the submission (e.g., sections or figure legends), or explain why this information doesn't apply to your submission:

In each figure legend, Materials and Methods we describe sample size and statistical methods used. No statistical methods were used to predetermine sample size. For cell based experiments with low variability, three independent experiments were performed. For animal based trial, at least 3-5 animals were included for different time points in at least three independent experiments.

Replicates

- You should report how often each experiment was performed
- You should include a definition of biological versus technical replication
- The data obtained should be provided and sufficient information should be provided to indicate the number of independent biological and/or technical replicates
- If you encountered any outliers, you should describe how these were handled
- Criteria for exclusion/inclusion of data should be clearly stated
- High-throughput sequence data should be uploaded before submission, with a private link for reviewers provided (these are available from both GEO and ArrayExpress)

Please outline where this information can be found within the submission (e.g., sections or figure legends), or explain why this information doesn't apply to your submission:

Sample sizes and experiment replicates were detailed in each figure legend. For cell based experiment with low variability (including in situ hybridization, immuno-staining, length measurement, cell counting, ChIP, western blot, qRT-PCR), at least three independent experiments were performed. For animal based trial, at least 3-9 animals were included for different time points in at least three independent experiments.

Statistical reporting

- Statistical analysis methods should be described and justified
- Raw data should be presented in figures whenever informative to do so (typically when N per group is less than 10)
- For each experiment, you should identify the statistical tests used, exact values of N, definitions of center, methods of multiple test correction, and dispersion and precision measures (e.g., mean, median, SD, SEM, confidence intervals; and, for the major substantive results, a measure of effect size (e.g., Pearson's r, Cohen's d)
- Report exact p-values wherever possible alongside the summary statistics and 95% confidence intervals. These should be reported for all key questions and not only when the p-value is less than 0.05.

Please outline where this information can be found within the submission (e.g., sections or figure legends), or explain why this information doesn't apply to your submission:

Gene expression data were normalized by using RMA algorithm in BioConductor software package. The k-Means Clustering algorithm and Eisen software tools were used to identify the distinct expression patterns of genes with Coefficient of Standard Deviation (C.S.D.) > 0.15 across the multiple zones. For each gene, the C.S.D. value was calculated with the formula: $\text{C.S.D.} = \frac{S}{\bar{X}}$, where S is the standard deviation and $\bar{X}$ is the mean expression level of the samples over the sectioned zones. The Gene Ontology analysis was performed for each cluster of genes by using the Gene Ontology database and the David Web Tools. For motif discovery, 2-kb DNA sequences upstream to the Transcription Start Site of the differentially expressed genes in each cluster were extracted for input to the HOMER motif analysis with the default program parameter setting. For the cell based and mouse work, statistical analyses used are detailed in the figure legends. Unpaired two-tailed Student's t-test was used to establish statistical significance. For growth analysis, two-tailed Mann-Whitney U-test was used. $p < 0.05$ was considered statistically significant.

(For large datasets, or papers with a very large number of statistical tests, you may upload a single table file with tests, Ns, etc., with reference to sections in the manuscript.)

Group allocation

- Indicate how samples were allocated into experimental groups (in the case of clinical studies, please specify allocation to treatment method); if randomization was used, please also state if restricted randomization was applied
- Indicate if masking was used during group allocation, data collection and/or data analysis

Please outline where this information can be found within the submission (e.g., sections or figure legends), or explain why this information doesn't apply to your submission:

For mouse study, mutant and transgenic mice were identified by genotyping. The investigators were blinded to group allocation during data collection, but not blinded during data analysis.

Additional data files ("source data")

- We encourage you to upload relevant additional data files, such as numerical data that are represented as a graph in a figure, or as a summary table

- Where provided, these should be in the most useful format, and they can be uploaded as “Source data” files linked to a main figure or table
- Include model definition files including the full list of parameters used
- Include code used for data analysis (e.g., R, MatLab)
- Avoid stating that data files are “available upon request”

Please indicate the figures or tables for which source data files have been provided:

All raw microarray data are deposited in the GEO website (GSE99306) and the ATF4 and CHOP ChIP-sequencing datasets were downloaded from the GEO database (GSE35681). R Bioconductor, HOMER and Picard Toolkit were used for data processing and analyses. Further statistical analyses were conducted by R bioconductor and Excel. All relevant data have been listed in the figures, supplementary figures and table S1-6.
